# Chronic Kidney Disease Requiring Healthcare Services: A New Approach to Evaluate Epidemiology of Renal Disease

**DOI:** 10.1155/2014/268362

**Published:** 2014-11-20

**Authors:** Gianluca Trifirò, Janet Sultana, Francesco Giorgianni, Ylenia Ingrasciotta, Michele Buemi, Marco Muscianisi, Daniele Ugo Tari, Margherita Perrotta, Valeria Canale, Vincenzo Arcoraci, Domenico Santoro

**Affiliations:** ^1^Department of Clinical and Experimental Medicine, University of Messina and Policlinico Universitario, Via Consolare Valeria, 98125 Messina, Italy; ^2^Caserta-1 Local Health Service, 81100 Caserta, Italy

## Abstract

*Background.* Screening-based CKD estimates may not provide a sufficient insight into the impact of CKD on the use of healthcare resources in clinical practice. The aim of this study was to evaluate the epidemiology of “medicalized” CKD, that is, CKD requiring healthcare services, in an outpatient setting. *Design, Setting, Participants, and Measurements.* This is a retrospective, longitudinal population-based study conducted in a large general practice setting in Southern Italy (Caserta) using a healthcare database. Over 2006–2011, all patients with a CKD diagnosis, either through CKD-related indications of use associated with drug prescriptions or through CKD-related hospital discharge diagnoses/procedures, were identified using this database. The prevalence of “medicalized” CKD in the general population of Caserta was estimated by age, gender, and calendar year. *Results.* Overall, 1,989 (1.3%) patients with a diagnosis of CKD were identified from 2006–2011 in the Caserta general population. The one year prevalence increased from 0.9% in 2006 to 1.6% in 2011, which is much lower compared to previous screening-based studies. The prevalence was slightly higher in males and increased significantly with advancing age (in 2011, 0.2% in ≤44 years old versus 9.2% in >80 years old). *Conclusions.* The findings of this study suggest that, in the general population, the prevalence of “medicalized” CKD is lower compared to the screening-based CKD prevalence.

## 1. Background

The number of patients worldwide with chronic kidney disease (CKD) is continuously increasing. Although CKD has been a somewhat scientifically neglected chronic noncommunicable disease [1], the global burden of CKD has been found to increase year after year. The main driving factors behind this are the increasingly aged global population [2] and the worldwide epidemic of type 2 diabetes mellitus [3].

Prevalence studies have a particularly relevant role in healthcare planning since global healthcare resources are limited, while healthcare needs are constantly increasing [4]. Several epidemiological studies such as PREVEND (The Netherlands) [5], EPIRCE (Spain) [6], HUNT (Norway) [7], NHANES III (USA) [8], and INCIPE (Italy) [9] have explored the prevalence of the various stages of CKD in different countries. Depending upon the method for CKD identification or formula for estimating the glomerular filtration rate (GFR), the race (Caucasian, Afro-American or Asian, etc.), or the setting, the prevalence of different CKD stages is often comparable in these studies, ranging from 5.1 to 7.0% for stages 1 and 2 combined, from 4.5 to 5.3% for stage 3, and much lower for stages 4 and 5, from 0.1 to 0.4% (Table 1). Recently, data coming from Italian (CARHES) [10] and Chinese [11] studies that examined small samples of the general population found that prevalence of CKD is lower as compared to other countries, especially concerning CKD 3–5 stages. However, the real impact of CKD on healthcare systems has not been well determined because CKD studies generally do not specifically consider CKD cases that are allocated healthcare resources. In particular, the attention should be focused not only on the screening-based prevalence of CKD, but on the CKD populations that require the use of resources of the healthcare systems directly. For this reason, we explored the prevalence of CKD requiring drug prescriptions, hospital admissions, and procedures, that is, what we termed “medicalized” CKD, in a general population of Southern Italy using a claims database.

## 2. Methods

Data was extracted from the Arianna database during the years 2004–2011. This database was set up by the Caserta Local Health Unit in Southern Italy in the year 2000 and currently contains information on a population of 158,510 inhabitants (20% of population from Caserta catchment area), who are registered in the list of 123 general practitioners (GPs). During their daily routine care, GPs record and transfer anonymous patient clinical data to a central database through dedicated software.

The Arianna database contains data concerning all the drug prescriptions (and related indication of use) which are reimbursed by the National Health Service (NHS). This data can be linked to hospital discharge admissions and other registries through a unique patient identifier. Information on drugs is coded according to the Anatomical Therapeutic Chemical classification system (ATC), while indications for use and hospital discharge diagnoses/procedures are coded by the ninth edition of International Classification of Diseases-Clinical Modification (ICD-9 CM). Quality checks on the data are routinely carried out. Arianna database has been previously demonstrated as a valid source for epidemiological research [16–19].

We identified CKD patients, searching for the following specific renal diseases-related codes among either primary/secondary causes of hospital admission or indication of use associated to prescribed drugs: 250.4 (diabetes with renal manifestations), 285.21 (anemia in chronic kidney disease), 583^*^ (nephritis and nephropathy, not specified as acute or chronic), 585^*^ (chronic kidney disease), 586^*^ (renal failure, unspecified), 403^*^ (hypertensive chronic kidney disease), and 404^*^ (hypertensive heart and chronic kidney disease). Patients with ICD-9 codes 583^*^ or 586^*^ were considered as CKD patients only if these codes were repeated more than twice to prevent misclassifying acute renal disease as CKD. We identified CKD also in the presence of multiple registration of renal dialysis among procedures over time.

We calculated the one year prevalence of “medicalized” CKD, overall, by age groups and by sex, over the years 2006–2011, with the years 2004-2005 being considered as run-in period to characterize the patients. For each observation year, the CKD prevalence was calculated by dividing the number of patients with CKD diagnosis by the number of subject who were registered in the GPs' lists.

## 3. Results

Out of almost 160,000 persons from a general population of Southern Italy in the years 2006–2011, we identified 1,989 patients (1.3%) with a diagnosis of CKD requiring drug prescriptions for renal disease-related indications of use or hospital admission/procedures (Table 2). Specifically, 1,151 (58%) CKD patients were identified through primary or secondary hospital discharge diagnoses/procedures, while 838 (42%) were identified through prescriptions issued for CKD-related indications. Of the latter, 213 (11%) patients were prescribed allopurinol, 94 (4.7%) furosemide, 63 (3.2%) polystyrene sulfonate, and 56 (2.8%) calcitriol with a CKD-related indication of use, as defined by ICD-9 codes.

**Figure 1:**
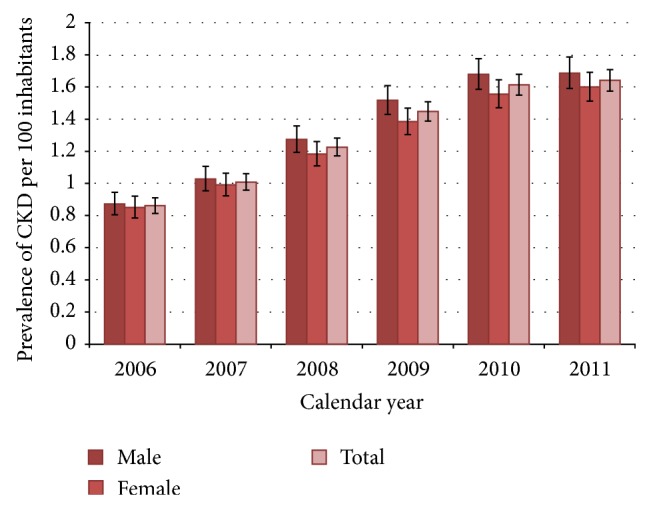
Annual prevalence (%) of “medicalized” chronic kidney disease in general population of Caserta in the years 2006–2011, overall, and stratified by sex. The bars on the columns represent the 95% confidence intervals for proportions.

**Figure 2:**
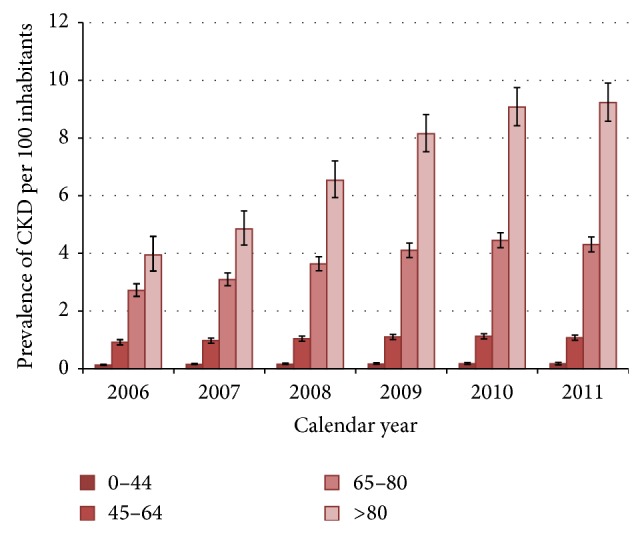
Annual prevalence (%) of “medicalized” chronic kidney disease in the Caserta general population in the years 2006–2011, stratified by age groups. The bars on the columns represent the 95% confidence intervals for proportions.

The one year prevalence of CKD increased over the study years from 0.9% in 2006 to 1.6% in 2011 (Figure 1). In general, the prevalence was slightly higher in males (males/females: 1.1) and increased significantly with advancing age from 0.2% in <45 years old patients to 9.2% in patients 80 years and over in 2011 (Figure 2). While the prevalence of CKD in patients over 80 years increased dramatically over the observation period, from 3.9 to 9.2 cases per 100 inhabitants from 2006 to 2011, the prevalence of CKD in the other age groups remained much more stable over the years, resulting in a widening gap between CKD patients over 80 and patients under 80.

## 4. Discussion

Several investigations have previously explored the epidemiology of CKD, mostly through screening of the general population or retrospective evaluation of GPs' electronic medical records, reporting heterogeneous results. The difference in CKD prevalence documented in various studies may be related to different methods of estimated creatinine clearance (eCrCl) MDRD (modification of diet in renal disease equation) as compared to CKD-EPI (chronic kidney disease epidemiology collaboration equation) [20]. It is known that CKD-EPI produces higher GFR and lower CKD estimates [21]. Another reason for the observed differences in CKD prevalence is the fact that some studies screened specifically frailer target groups, such as patients with diabetes and hypertension or elderly populations, leading to an overestimation of the true CKD prevalence in the general population [22]. In the United Kingdom, patients are selected for CKD screening programs using an existing laboratory database. With this approach only patients with elevated serum creatinine or eGFR < 60 mL/min are selected, thus precluding the identification of CKD patients at early stages (1 and 2) [23]. A better way of identifying those patients would be to select them by screening urine for a positivity using a dipstick test or using the albumin/creatinine ratio (ACR) [24]. Data from the third National Health and Nutrition Examination Survey showed that up to 11% of the general adult population could have some degree of CKD, which consisted of more than 8 million individuals with glomerular filtration rates of less than 60 mL per minute [8]. However, all the abovementioned screening studies, which are based on single measurement of serum creatinine or eClCr, are subject to variation owing to differences in calibration systems across different laboratories [25].

More importantly, the epidemiology of CKD based on screening studies of small samples of the general population and retrospective evaluation of GPs' records do not allow the evaluation of the impact on actual use of healthcare services of CKD patients. The assessment of disease burden by quantifying “medicalized CKD” in combination with traditional screening-based methods may allow more accurate healthcare cost estimations for health care providers as “medicalized” CKD estimates are based on the actual use of healthcare resources.

The approach used in this study may be considered innovative. The prevalence of CKD was estimated on the basis of use of healthcare services such as CKD-related hospital admissions/procedures and drug prescriptions issued for CKD-related indications. The overall estimated prevalence of CKD in our study is significantly lower as compared to previous epidemiologic investigations. The patients identified using the claims database are likely to be mostly affected by CKD stages 4 and 5 and, to a lesser extent, 3. In line with this hypothesis, our estimates are rather comparable to other studies when restricting the analysis to those CKD stages.

Some limitations warrant caution. Information on indication of use related to drugs being dispensed directly in hospital (e.g., epoetin, paricalcitol) was not available in Arianna database, which may have slightly underestimated the prevalence of “medicalized” CKD. However, data on the use of CKD-related hospital admissions/procedures is linked to the Arianna database, minimizing the likelihood of such an underestimation. Despite the existence of 5-digit CKD stage-specific ICD-9 codes, CKD was mostly coded using non-stage-specific 3-digit codes in the Arianna database, thus preventing the evaluation of the prevalence of CKD for each stage.

Another potential limitation in the estimation of CKD prevalence by identifying “medicalized CKD” is that ICD codes and drug prescription-guided CKD diagnosis may be influenced by CKD awareness. As a study conducted in the same geographical area suggests, awareness of CKD is very low among patients and general practitioners [26]. Indeed, the fact that CKD prevalence in our study almost doubled from 2006 to 2011 in a small catchment area may indirectly indicate an increased awareness in assigning CKD codes at hospital discharge or a more liberal prescription of drugs for CKD-related indications, rather than an effective increase in the prevalence of CKD. Nevertheless, this is still CKD which impacts on healthcare resources. Finally, the small size of the catchment area also represents a limitation where generalizability is concerned.

In conclusion, the main results of this study demonstrate that within the general population the prevalence of CKD requiring use of healthcare resources is lower as compared to the overall screening-based CKD prevalence. From a public health perspective, the epidemiologic evaluation of CKD requiring the use of healthcare resources; that is, “medicalized” CKD may be, in addition to traditional screening-based CKD epidemiology, valuable in providing a perspective useful to the allocation of healthcare budgets.

## Figures and Tables

**Table 1:** Overall and stage-specific CKD estimates from previous epidemiologic investigations worldwide.

Author, date	Setting-age	Country	*N*	Race	CKD Stage	Prevalence%	Stage 1	Stage 2	Stage 3	Stage 4	Stage 5	Method	Albuminuria
Coresh et al., 2003 [8]	General population; 18–95 years	USA	15,625	Afro-American	1–5	11	3.3	3	4.3	0.2	0.2	MDRD	No
Holmen et al., 2003 [12]	General population; >20 years	Norway	65,181	Caucasian	1–4	11.2	3.1	3.4	5.3	0.4	NA	MDRD	Yes
De Zeeuw et al., 2005 [5]	General population; 28–75 years	The Netherlands	3,432	Caucasian	1–5	10.6	1.3	3.8	5.3	0.1	0.1	cCrCl	Yes
Viktorsdottir et al., 2005 [13]	General population; 33–85 years	Iceland	19,381	Caucasian	1, 3-4	>9.2	1.6	NA	7.4	0.2	NA	MDRD; C-G	Yes
Cirillo et al., 2006 [14]	General population; 18–95 years	Italy	4,574	Caucasian	3–5	6.6 M—6.2 F		NA	NA	NA	NA	MDRD	Yes
Gambaro et al., 2010 [9]	General population; >40 years	Italy	3,870	Caucasian	1–4	12.7 (13.2 M—12.2 F)	1.7	4.3	6.4	0.3	NA	CKD-EPI	Yes
Gonz$\overset{´}{\text{a}}$lez et al., 2010 [6]	General population; >20 years	Spain	2,746	Caucasian	1–5	12.5	1.0	1.3	6.5	0.3	0.03	MDRD	Yes
de Nicola et al., 2011 [10]	General population; 35–79 years	Italy	3,559	Caucasian	1–5	7.1 (7.5 M—6.5 F)	2.6	1.5	2.6	0.2	0.1	CKD-EPI	Yes
Capuano et al., 2012 [15]	General population; 25–74 years	Italy	1,200	Caucasian	1–5	5.9 M—3.9 F (1998-1999) 6.2 M—9.0 F (2008-2009)	NA	NA	NA	NA	NA	C-G	No
Zhang et al., 2012 [11]	General population; 18–95 years	China	47,204	Chinese	1–5	10.8	5.7	3.4	1.6	0.1	0.03	MDRD	Yes

C-G: Cockroft-Gault equation; cCrCl: calculated creatinine clearance; CKD-EPI: chronic kidney disease epidemiology collaboration equation; CKD: chronic kidney disease; F: females; *N*: number; NA: not available; M: males; MDRD: modification of diet in renal disease equation.

**Table 2:** Healthcare resources used for the identification of first CKD diagnosis.

Healthcare resources	CKD patients
	*N* = 1,989 (%)
CKD-related hospital discharge diagnoses^*^	**1,151 (57.9)**
Primary diagnosis	258 (13.0)
Secondary diagnosis	867 (43.6)
Procedures	39 (2.0)
CKD-related conditions as indication of use for drug prescriptions	**838 (42.1)**
Allopurinol	213 (10.7)
Furosemide	94 (4.7)
Polystyrene sulfonate	63 (3.2)
Calcitriol	56 (2.8)
Ramipril	29 (1.5)
Metformin and sulfonamides	28 (1.4)
Acetylsalicylic acid	24 (1.2)
Metformin	22 (1.1)
Electrolytes solutions	19 (1.0)
Glimepiride	16 (0.8)
Ferrous sulfate	12 (0.6)
Torasemide	12 (0.6)
Amlodipine	11 (0.6)
Simvastatin	10 (0.5)
Bisoprolol	9 (0.5)
Nebivolol	9 (0.5)
Losartan	9 (0.5)
Atorvastatin	9 (0.5)
Spironolactone	8 (0.4)
Ramipril plus diuretics	8 (0.4)
Others^**^	177 (33.2)

^*^Categories of discharge diagnoses and procedures are not mutually exclusive as in some patients CKD-related discharge diagnosis and procedures may have been registered at the same time.

^**^Other drugs which account for less than 0.4% of first CKD diagnosis identification.

## Abbreviations

ACR: Albumin/creatinine ratio
ATC: Anatomical Therapeutic Chemical classification system
CARHES: Cardiovascular risk in Renal Patients of the Italian Health Examination Survey
CKD: Chronic kidney disease
CKD-EPI: Chronic Kidney Disease Epidemiology Collaboration equation
eCrCl: Estimated creatinine clearance
EPIRCE: Estudio Epidemiológico de la Insuficiencia Renal en España
GFR: Glomerular filtration rate
HUNT: Helseundersøkelsen i Nord-Trøndelag
ICD-9 CM: International Classification of Diseases - Clinical Modification
INCIPE: Initiative on Nephropathy, of relevance to public health, which is chronic, possibly in its initial stages, and carries a potential risk of major clinical end-points
MDRD: Modification of diet in renal disease equation
NHANES III: National Health and Nutrition Examination Survey
NHS: National Healthcare Service
PREVEND: Prevention of Renal and Vascular End-Stage Disease Intervention Trial.
